# USP5 Promotes Metastasis in Non-Small Cell Lung Cancer by Inducing Epithelial-Mesenchymal Transition *via* Wnt/β-Catenin Pathway

**DOI:** 10.3389/fphar.2020.00668

**Published:** 2020-05-08

**Authors:** Sudong Xue, Wei Wu, Ziyan Wang, Guangxian Lu, Jiantong Sun, Xing Jin, Linjun Xie, Xiaoyu Wang, Caihong Tan, Zheng Wang, Wenjuan Wang, Xinyuan Ding

**Affiliations:** ^1^Department of Pharmacy, the Affiliated Suzhou Hospital of Nanjing Medical University, Suzhou, China; ^2^Department of Pharmacy, Zhongshan Hospital, Fudan University, Shanghai, China; ^3^Department of Pharmacy, The Affiliated Hospital of Jiangsu University, Zhenjiang, China; ^4^Comprehensive Breast Health Center, Ruijin Hospital, Shanghai Jiao Tong University School of Medicine, Shanghai, China; ^5^Department of Pharmacy, The Children's Hospital of Soochow University, Suzhou, China

**Keywords:** non-small cell lung cancer, metastasis, epithelial-to-mesenchymal transition, ubiquitin-specific protease 5 (USP5), β-catenin

## Abstract

Ubiquitin-specific protease 5 (USP5) is a deubiquitinating enzyme that functions as an oncoprotein in a variety of human cancers. However, the expression and role of USP5 in the metastasis of non-small cell lung cancer (NSCLC) have not been addressed. In this study, we examined the expression and prognostic significance of USP5 in NSCLC. The results revealed that USP5 was overexpressed and correlated with metastasis and overall survival in NSCLC tissues. A further *in vitro* study revealed that the levels of USP5 protein in NSCLC cells were associated with epithelial–mesenchymal transition (EMT) markers. Furthermore, USP5 overexpression significantly enhanced, whereas USP5 silencing significantly decreased the expression of EMT proteins and migration and invasion of NSCLC cells. In addition, the results from western blotting demonstrated that USP5 regulated EMT *via* the Wnt/β-catenin signaling pathway. Further immunohistochemical analysis revealed that USP5 was significantly associated with the expression of β-catenin and EMT markers in NSCLC tissues. Overall, USP5 upregulation is associated with tumor metastasis and poor prognosis in patients with NSCLC. USP5 promotes EMT and the invasion and migration of NSCLC cells. Therefore, USP5 may serve as a novel prognostic biomarker and provide a potential target for the treatment of metastasis in NSCLC.

## Introduction

Lung cancer is the most commonly diagnosed malignancy and a prime leading cause of cancer deaths worldwide (Bray et al., 2018). Non-small cell lung cancer (NSCLC), the main pathological type of which is adenocarcinoma, accounts for about 85% of all lung cancers (Zhu et al., 2017). Despite advances in the treatment of NSCLC, distant metastasis remains the main factor contributing to disease-associated death (Chaffer and Weinberg, 2011). Therefore, a better understanding of the mechanisms by which tumor metastasis is regulated and the identification of new targets are urgently needed.

Epithelial–mesenchymal transition (EMT) is a process with particular phenotypic changes, through which epithelial cells lose their cell–cell adhesion and cell polarity, increase the expression of mesenchymal cell markers and migratory capacity, resist apoptosis, and produce extracellular matrix components (Thiery et al., 2009; Jiang et al., 2019). It is well known that EMT plays important roles in a variety of biological processes, for instance, wound healing, embryonic development, fibrosis, and organ formation (Sung et al., 2016). Recent cancer studies have revealed that EMT, which is essential for the transformation of early-stage tumors into aggressive malignancies, is an early event in tumor metastasis (Kang and Massague, 2004). Moreover, numerous studies have shown that signaling pathways such as phosphatidylinositol-3-kinase/AKT, nuclear factor-κB, mitogen-activated protein kinase, Notch, transforming growth factor (TGF)-β, and Wnt/β-catenin, among others, play crucial roles in EMT-related invasion and metastasis (Weis and Cheresh, 2011; Katsuno et al., 2013).

Ubiquitination and deubiquitination are the two main types of posttranslational modifications, which rely on ubiquitin ligases and deubiquitinating enzymes (DUBs) to maintain protein homeostasis during biological processes and often result in disease (Reyes-Turcu et al., 2009). We previously reported that ubiquitin C-terminal hydrolase L1 (UCHL1), a member of DUBs, is associated with favorable prognosis in neuroblastoma (Gu et al., 2018a) and is critical for the survival and immunosuppressive function of tumor-associated immune cells (Gu et al., 2018b), suggesting that these DUBs are closely related with tumor development and progression. USP5, a member of the ubiquitin-specific protease (USP) family of DUBs, removes ubiquitin from the proximal end of unanchored polyubiquitin chains (Avvakumov et al., 2012). Increasing evidence indicates that USP5 functions as an oncoprotein in a variety of human cancers. For instance, USP5 promotes tumorigenesis and drug resistance through inhibiting the p14ARF-p53 signaling pathway in hepatocellular carcinoma (Liu et al., 2017). USP5 is overexpressed in colorectal cancer tissues and promotes colorectal cancer cell proliferation and resistance to chemotherapeutics (Xu et al., 2019). USP5 targets the transcription factor SLUG and then induces EMT in hepatocellular carcinoma cells (Meng et al., 2019). However, the precise role of USP5 in EMT in NSCLC and the underlying mechanism have not been explored.

In this study, we examined USP5 expression in patients with NSCLC and found a negative association between USP5 levels and tumor metastasis in patients with NSCLC. Overexpressing the USP5 protein enhanced, whereas repressing the USP5 protein impeded NSCLC cell migration, invasion, and EMT. We then discovered that β-catenin signaling activation, which plays vital roles in EMT, was restrained in USP5-knockdown NSCLC cells. Furthermore, we found that USP5 was associated with the expression of EMT markers and β-catenin in NSCLC tissues. These data demonstrate that USP5 plays an important role in promoting NSCLC metastasis and may be a promising pharmaceutical target for NSCLC metastasis.

## Materials and Methods

### Patients and NSCLC Specimens

Forty-seven primary NSCLC and adjacent normal tissue samples (1.5–2 cm away from the tumor site) were obtained after acquiring informed consent from patients in the Affiliated Hospital of Jiangsu University (Zhenjiang, China) between March 2012 and February 2014. Pathological diagnostic data of patients with NSCLC were assessed according to the Revised International System for Staging Lung Cancer. None of the patients had received either chemotherapy or radiotherapy before undergoing surgery. The recorded data of each patient were analyzed, and follow-up was performed through phone calls. All experimental protocols were approved by the Ethics Committee of the Affiliated Hospital of Jiangsu University and Medical College of Jiangsu University and were conducted in accordance with the Helsinki Declaration.

### Immunohistochemistry

Immunohistochemistry (IHC) staining was performed using a standard immunoperoxidase staining procedure to detect the levels of USP5 in paraffin-embedded NSCLC specimens. Rabbit anti-human USP5 antibody (Proteintech, Chicago, Illinois, USA) was employed as the primary antibody with a 1:300 dilution, followed by an anti-rabbit secondary antibody using the DAKO ChemMate™ Envision™ Detection Kit (DAKO A/S, Denmark). Positive staining for USP5 (in brown) was mainly localized in the cytoplasm. The expression status of each tissue sample was assessed according to a previously reported method (Wang et al., 2019b). H-scores were determined based on both the intensity and percentage of USP5-positive tumor cells. The staining intensity was scored based on four categories: negative (0), weak (1+), moderate (2+), and strong (3+). A 10-tiered scale (10 to 100%) was used to score the percentage of USP5-positive tumor cells. The H-score was calculated using the following formula: 1 × [percentage of cells staining weakly (+)] + 2 × [percentage of cells staining moderately (++)] + 3 × [percentage of cells staining intensely (+++)]. The overall score ranged from 0 to 300. For dichotomization, the cohort was divided into 2 subgroups based on the metastasis status; the optimal cutoff value was calculated in the R statistical environment using the “survival ROC” package and used to determine the degree of USP5 expression (USP5-high or -low) that was associated with metastasis (Chu et al., 2019).

### Validation of Human Datasets

To explore USP5 transcripts in NSCLC based on microarray studies, the tissue data were obtained from the R2 Genomics Analysis and Visualization Platform (http://r2.amc.nl) using the publicly available dataset, The Cancer Genome Atlas (TCGA) (lung adenocarcinoma, containing 515 samples). Of these, 509 samples with clear metastatic information were used. The Kaplan–Meier-plotter dataset (206031_s_at, http://kmplot.com/analysis) and GEPIA dataset (http://gepia.cancer-pku.cn) were utilized to analyze the correlation between USP5 expression and the prognosis of patients diagnosed with lung adenocarcinoma.

### Cell Culture and Transfection

The normal human lung fibroblast WI-38 and NSCLC (W-358, A549, H1299, and 95-D) cell lines were purchased from the American Type Culture Collection and cultured under recommended conditions. HEK293T cells were used to produce recombinant lentiviral stocks by co-transfecting the transfer vector, packaging plasmids pMD2.G and pSPAX2 (Addgene, Cambridge, USA), using Lipofectamine 2000 (Invitrogen, Carlsbad, CA, USA) according to the manufacturer's protocol. Lentivirus carrying the short-hairpin RNA (shRNA) were prepared and added to NSCLC cells in the presence of polybrene (Life Technologies, Darmstadt, Germany) according to the manufacturer's protocol. The medium was replaced with fresh culture medium after 24 h and expanded for follow-up experiments.

### Cell Migration and Invasion Assays

The protocol was performed as described (Wang et al., 2019a). For the wound-healing assay, the H1299 and 95-D cells were grown in 6-well plates. When a confluent layer was achieved, a sterile 200-μl pipette tip was used to create a scratch on the cell monolayer and then further incubated with fresh culture medium containing 1% fetal bovine serum. The status of the scratch wound closure was observed after 24 h by considering the distance ratio of the scratch wound at 24 h to the scratch wound at 0 h. The migration of NSCLC cells was measured by the cells that moved into the scarped site using an inverted phase-contrast microscope.

For the invasion assays, H1299 and 95-D cells subjected to different treatments were added in top-chamber inserts coated with matrigel (BD Biosciences, San Jose, CA, USA). The bottom chamber was filled with the complete medium. After being cultured at 37°C for 24 h, cells from the interiors of the inserts were removed, and the membranes were washed three times with phosphate-buffered saline (PBS), fixed in 4% paraformaldehyde, and stained with crystal violet (Sangon Biotech, Shanghai, China). The cells were counted by photographing the membrane through the microscope.

### Real-Time Polymerase Chain Reaction Assays

Total RNA was extracted using TRIzol (Invitrogen, Carlsbad, CA, USA) and reverse-transcribed into complementary DNA (cDNA) using a PrimeScript™ RT reagent kit (Takara Bio, Tsu, Japan). Real-time polymerase chain reaction (PCR) assays were performed using a ViiA 7 Real-time PCR System (Bio-Rad, Hercules, CA, USA) with SYBR Green reagent (Roche Applied Science, Natley, NJ, USA). Both procedures were performed in accordance with the manufacturer's instructions. The relative *USP5* and other mRNA expression levels were determined and normalized to the level of glyceraldehyde 3-phosphate dehydrogenase (*GAPDH*). The sequences of the primers used in this study are listed in **Table S1**.

### Western Blot Assay

Western blot assays were conducted as previously described (Xu et al., 2014). The proteins were extracted using a radioimmunoprecipitation assay buffer (Sangon Biotech), separated by sodium dodecyl sulfate polyacrylamide gel electrophoresis, and transferred to polyvinylidene difluoride membranes. The membranes were then blocked for 1 h and incubated with the indicated antibody in PBS plus 5% bovine serum albumin overnight at 4 °C. After washing with PBS plus 0.1% Tween-20, the membranes were incubated with horseradish peroxidase-conjugated goat anti-mouse or anti-rabbit immunoglobulin G (Sangon Biotech) for 1 h. After subsequently washing, the immunoreactive bands were visualized using the Tannon 2500 imaging system, and the densitometry of the bands was analyzed using ImageJ software. Primary anti-human antibody against USP5 was purchased from Abcam (Cambridge, MA, USA), and E-cadherin, N-cadherin, vimentin, β-catenin, non-phospho (Active) β-catenin (Ser33/37/Thr41), glycogen synthase kinase-3β (GSK-3β), phospho-GSK-3β (Ser9), ubiquitin, and GAPDH were all purchased from Cell Signaling Technology (CST, Danvers, MA, USA).

### Ubiquitination and Protein Stability Assay

Transfected cells were treated with a proteasome inhibitor MG132 (MedChemExpress, Monmouth Junction, NJ, USA), and whole-cell extracts were then immunoprecipitated with anti-β-catenin antibody and analyzed by western blotting for anti-ubiquitin antibody. Transfected cells treated with MG132 were harvested to detect β-catenin and active β-catenin protein stability by western blot.

### Immunofluorescence Staining

Cells were grown on glass coverslips and then fixed by 4% paraformaldehyde for 20 min. Following the PBS wash, cells were permeabilized using 0.1% Triton X-100, then blocked with bovine serum albumin (3%), and further incubated overnight at 4°C with primary antibody USP5 (CST, Danvers, MA, USA). Thereafter, Alexa Fluor 488 (Invitrogen) was added as a fluorescent conjugated secondary antibody for 1 h at 37 °C in the dark. DAPI (Sigma Aldrich, St Louis, MO, USA) was used to stain the nuclei of the cells. Finally, the coverslips were mounted onto slides with fluorescent mounting medium and immediately observed using a fluorescence microscope (Carl Zeiss, Jena, Germany).

### Lentiviral Vector Construction

Silencing of gene expression was achieved using shRNA technology. shRNAs targeting human *USP5* (sh*USP5*), 5'-CCGGGACCACACGATTTGCCTCATTCTCGAGAATGAGGCAAATCGTGTGGTCTTTTTG-3' (sh*USP5*-1) and 5'- CCGGCCTGTCTGTAAGGAGACTTTGCTCGAGCAAAGTCTCCTTACAGACAGGTTTTTG-3' (sh*USP5*-2), and scrambled control (shNC), 5'-CCGGCCTAAGGTTAAGTCGCCCTCGCTCGAGCGAGGGCGACTTAACCTTAGGTTTTTG-3' (synthetized by Sangon Biotech), were cloned into pLKO.1 puro vector (Addgene, Cambridge, USA). H1299 and 95-D cells were infected with high-titer lentiviral stocks expressing shRNA (-shNC or -sh*USP5*).

Full-length *USP5* cDNA were synthesized using Genscript (Nanjing, China). These cDNAs were subcloned into pcDNA3.1(+) vectors containing an N-terminal C-Myc epitope tag. Cells were transfected with pcDNA3.1(+) (-vec) or pcDNA3.1(+) containing USP5 (-*USP5*) using Lipofectamine 2000 according to the manufacturer's protocol.

### Statistical Analysis

All measurement data were presented as means ± standard errors of the mean. Statistical significance of continuous variables was evaluated based on the Mann–Whitney test and analysis of variance using GraphPad Prism (version 6.0) and Statistical Package for Social Science software (version 22.0). Linear regression analyses were performed to examine the interaction between USP5 protein levels and EMT protein levels. Correlations between USP5 expression and clinicopathologic patterns were assessed based on the χ^2^ test or Fisher's exact test. Survival analysis was assessed based on Kaplan–Meier analysis. *p* < 0.05 was considered statistically significant.

## Results

### High USP5 Expression Was Associated With Metastasis of NSCLC

**Figure 1:**
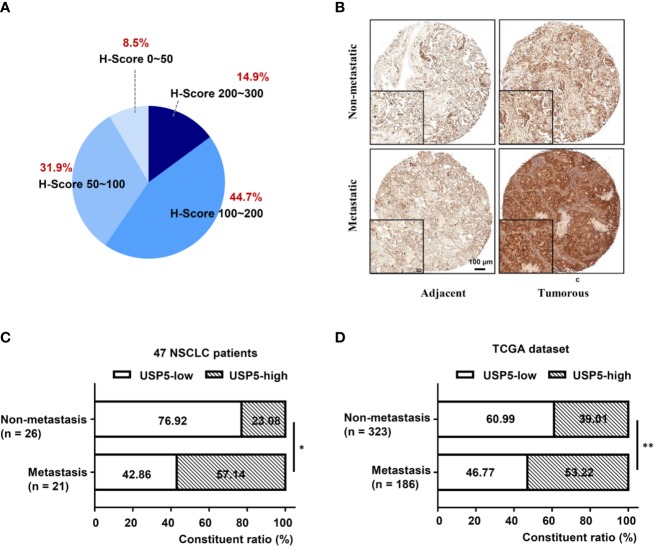
High USP5 expression was associated with metastasis in NSCLC. **(A)** The proportions of scores (0–300) of IHC staining of USP5 expression in 47 NSCLC samples. **(B)** Representative images showing different expression levels. Black bar, 100 μm. Comparison of metastatic status in 47 patients with NSCLC **(C)** and 509 lung adenocarcinoma samples from TCGA dataset **(D)** with different levels of USP5 expression. Statistical analysis was performed using the χ^2^ test. ^*^p < 0.05, ^**^p < 0.01. USP5, ubiquitin-specific protease 5; NSCLC, non-small cell lung cancer; IHC, immunohistochemistry; TCGA, The Cancer Genome Atlas.

IHC tests were performed to examine the expression of USP5 in NSCLC tissues and adjacent normal tissues. As shown in **Figure 1A**, the IHC scores of USP5 in different samples from patients with NSCLC ranged from 0 to 300. Adjacent normal tissues revealed weak or partly moderate USP5 staining restricted to the basal layers, whereas USP5 staining in NSCLC tissues was considerably stronger (**Figure 1B**). Patients with NSCLC were classified into UCHL1-high or UCHL1-low subgroups based on the cutoff value (125) of the IHC score. As shown in **Table 1**, there were no significant associations among UCHL1 expression and clinicopathological parameters, such as age at diagnosis, stage, histological type, and degree of tumor differentiation. However, USP5 expression levels were significantly correlated with tumor metastasis in patients with NSCLC (**Figure 1C**). This result was reinforced by the data of 509 patients with lung adenocarcinoma from TCGA dataset (**Figure 1D**). The clinicopathologic parameters of these patients are shown in **Table S2**. These data suggested that USP5 may serve a vital role in the metastasis of NSCLC.

**Table 1:** Associations among USP5 expression and clinicopathological characteristics in 47 patients with NSCLC.

Clinical pathologic characteristics		Case No.	USP5 expression		*p*
			Low	high	
Total cases		47	29	18	
Gender					
	Male	26	15	11	0.5292
	Female	21	14	7	
Age (years)					
	< 60	20	10	10	0.1555
	≥60	27	19	8	
Histological type					
	SCC	17	9	8	0.3523
	ADC	30	20	10	
Differentiation					
	Well	7	2	5	0.0937
	Moderate	34	24	10	
	Poor	6	3	3	
TNM stage					
	I-II	27	18	9	0.4159
	III-IV	20	11	9	
Metastasis					
	Metastasis	26	20	6	0.0169^*^
	Non-metasitasis	21	9	12

USP5, ubiquitin-specific protease 5; NSCLC, non-small cell lung cancer; SCC, squamous cell carcinoma; ADC, adenocarcinoma. Analysis by χ^2^ test or Fisher's exact test, *p < 0.05 value is set for highly significant difference.

### USP5 Was a Potential Prognostic Factor in NSCLC

**Figure 2:**
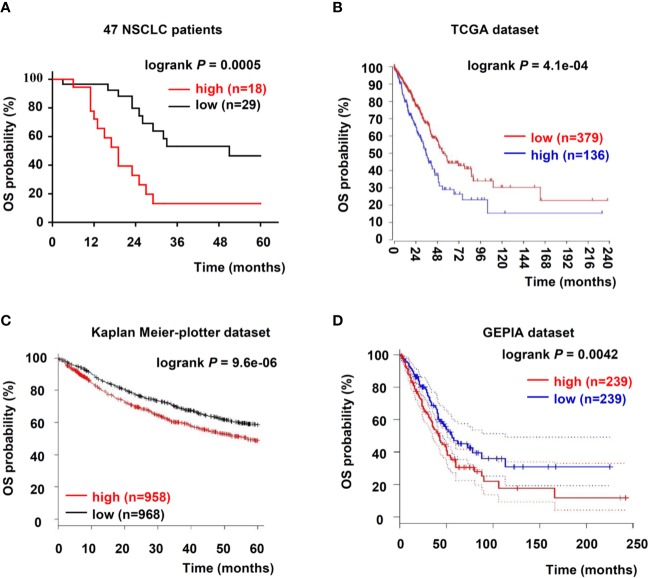
Upregulation of USP5 correlated with poor prognosis of patients with NSCLC. **(A)** Kaplan–Meier analysis of OS in 47 patients with NSCLC based on USP5 expression. OS was also determined according to USP5 expression in NSCLC samples from three public datasets: TCGA **(B)**, Kaplan–Meier-plotter **(C)**, and GEPIA **(D)**. Statistical analysis was carried out using a log-rank test. USP5, ubiquitin-specific protease 5; NSCLC, non-small cell lung cancer; OS, overall survival; TCGA, The Cancer Genome Atlas.

To further evaluate USP5 as a potential prognostic factor in NSCLC, the relationship between USP5 and survival rate in patients with NSCLC was analyzed; the data from 47 patients with NSCLC and TCGA dataset revealed that USP5 expression was negatively associated with the 5-year overall survival (OS) rate of NSCLC and the 20-year OS rate of lung adenocarcinoma, respectively (**Figures 2A, B**).

Furthermore, we reanalyzed two independent patient datasets (Kaplan–Meier-plotter and GEPIA) to examine the correlation between the mRNA expression of USP5 and survival rate of patients with NSCLC. In the Kaplan–Meier-plotter dataset, patients with low USP5 mRNA levels showed better 5-year OS (n = 1926, **Figure 2C**). Based on Kaplan–Meier analysis, we also confirmed that low USP5 expression was prognostic for favorable outcomes in the GEPIA dataset (20-year OS, n = 478, **Figure 2D**). Taken together, our data and three independent datasets indicated that USP5 was a potential prognostic marker in NSCLC.

### High USP5 Expression Was Correlated With EMT, Invasion, and Migration in NSCLC Cells

**Figure 3:**
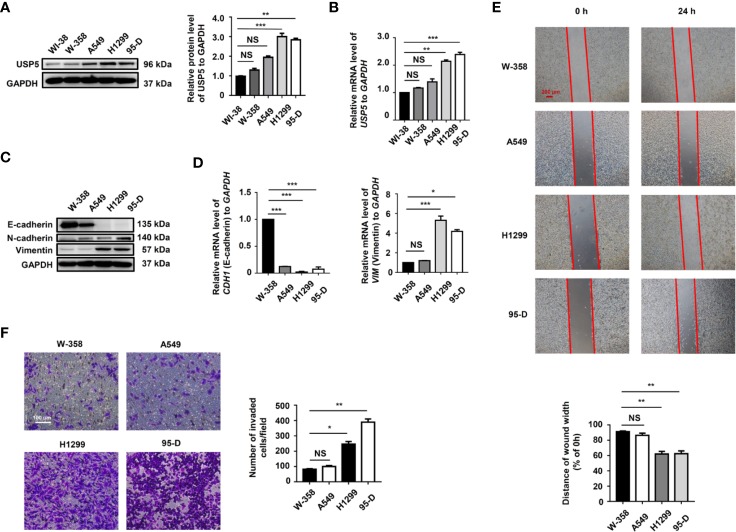
High USP5 expression was correlated with EMT, invasion, and migration in NSCLC cells. **(A)** Western blot and **(B)** real-time PCR analysis of USP5 expression in NSCLC cell lines and normal lung fibroblast cells. GAPDH was used as an internal normalization control. The protein **(C)** and mRNA **(D)** levels of EMT markers were analyzed in NSCLC cells. **(E)** Migration of NSCLC cells was determined based on a wound-healing assay (top panel). Red bar, 200 μm. Statistical results were determined using an ANOVA (bottom panel). **(F)** Cell invasion was determined based on a transwell invasion assay (left panel) and assessed using an ANOVA (right panel). All results were obtained in five independent experiments (n = 5). NS = no statistical significance, ^*^p < 0.05, ^**^p < 0.01, ^***^p < 0.001. USP5, ubiquitin-specific protease 5; EMT, epithelial–mesenchymal transition; NSCLC, non-small cell lung cancer; PCR, polymerase chain reaction; GAPDH, glyceraldehyde 3-phosphate dehydrogenase; ANOVA, analysis of variance.

We examined the level of USP5 expression in several NSCLC cell lines. The results showed that compared to those in normal lung fibroblast cells, the protein and mRNA levels of USP5 were significantly increased in NSCLC cells (**Figures 3A, B**). Surprisingly, higher USP5 expression was also found in H1299 and 95-D cells compared with that in W358 and A549 cells. H1299 and 95-D cells had a higher EMT profile than that in W358 and A549 cells, as described by the lower expression of the epithelial marker E-cadherin and higher expression of the mesenchymal markers N-cadherin and vimentin in H1299 and 95-D cells at both the mRNA and protein levels (**Figures 3C, D**). We also analyzed the correlation between the EMT profile and USP5 expression and showed that USP5 expression had a significant negative correlation with E-cadherin expression and significant positive correlations with N-cadherin and vimentin expression (**Figure S1**). As expected, greater abilities of both invasion and migration in H1299 and 95-D cells than those in W-358 and A549 cells was shown by performing invasion and migration assays (**Figures 3E, F**). All these results indicated that USP5 expression was positively correlated with EMT, invasion, and migration in NSCLC cells.

### USP5 Promoted EMT, Invasion, and Migration in NSCLC Cells

**Figure 4:**
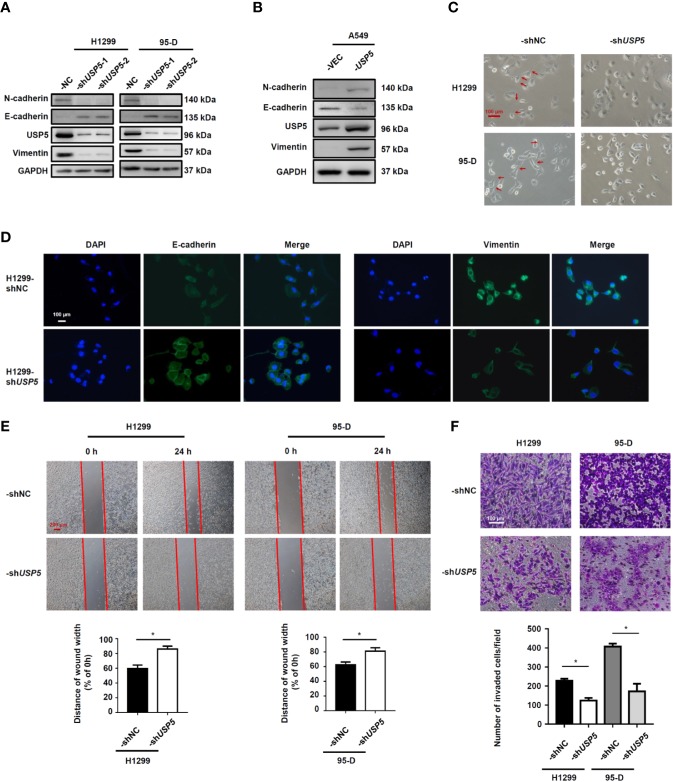
USP5 induces EMT and promotes migration and invasion in NSCLC cells. NSCLC cells were transfected with -sh*USP5* (-sh*USP5*-1 and -sh*USP5*-2) or -shNC lentiviruses **(A)** and -*USP5* or -VEC plasmids **(B)**; the protein expressions of EMT markers were detected by western blotting. **(C)** Representative photomicrographs of the four stably transfected NSCLC cells. Red bar, 100 μm. **(D)** The epithelial phenotype marker E-cadherin (green) and mesenchymal marker vimentin (green) were detected by IF staining. Nuclei were stained with DAPI (blue). White bar, 100 μm. **(E)** Cell migration was determined based on a wound-healing assay (top panel). Red bar, 200 μm. Statistical results were determined using the Mann–Whitney test (n = 5, bottom panel). **(F)** Cell invasion was determined based on a transwell invasion assay (top panel) and analyzed using the Mann–Whitney test (n = 5, bottom panel). ^*^p < 0.05, vs. control group. USP5, ubiquitin-specific protease 5; EMT, epithelial–mesenchymal transition; NSCLC, non-small cell lung cancer; IF, immunofluorescence.

As USP5 expression was significantly elevated in metastatic NSCLC tissues, we performed knockdown of USP5 expression with shRNA (-sh*USP5*-1 and -sh*USP5*-2) lentiviruses in both H1299 and 95-D cells, which highly expressed USP5. shNC NSCLC cells showed stable high levels of USP5 expression, whereas both -sh*USP5* cells showed a significant decrease by more than 80% correspondingly (**Figure 4A**). Meanwhile, -sh*USP5* cells resulted in significant upregulation of E-cadherin, whereas N-cadherin and vimentin were down-regulated. The sh*USP5*-1 vector was selected to knockdown the USP5 protein subsequently. Furthermore, when *USP5* was introduced into A549 cells by transfecting -*USP5* plasmids, designated as A549-*USP5*, the levels of E-cadherin reduced, whereas the levels of N-cadherin and vimentin elevated (**Figure 4B**). These data indicated that USP5 effectively promoted EMT in NSCLC cells.

In addition, the representative photomicrographs showed that sh*USP5* stimulated the cell phenotype to change from a mesenchymal phenotype to an epithelial phenotype (**Figure 4C**). Meanwhile, the IF analysis illustrated that compared with that in H1299-shNC cells, the H1299-sh*USP5* cells exhibited lower expression of vimentin protein and higher expression of E-cadherin protein in the cytoplasm (**Figure 4D**). To determine whether USP5 had the potential to promote migration and invasion, wound-healing and transwell invasion assays were performed using NSCLC cells. In the cell migration experiment, the widths of wound scratches in -sh*USP5* of H1299 and 95-D cells were much wider than those presented in the control group at 24 h, indicating that USP5 knockdown had the potential to inhibit the migration of NSCLC cells (**Figure 4E**). In the cell invasion assay, the numbers of invaded cells decreased in the -sh*USP5* group compared with those in the control group (**Figure 4F**). These results were reinforced by migration and invasion assays using A549-*USP5* cells (**Figure S2**). It was suggested that USP5 promoted EMT, invasion, and migration in NSCLC cells.

### USP5 Regulated EMT *via* the Wnt/β-Catenin Signaling Pathway

**Figure 5:**
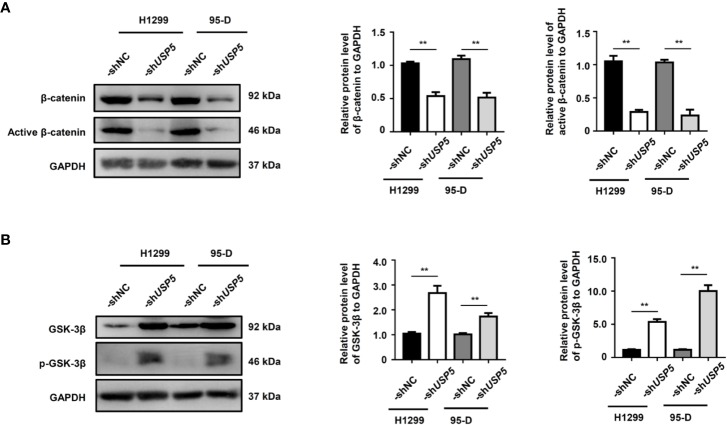
USP5 promotes EMT through the regulation of GSK-3β/β-catenin. The levels of **(A)** β-catenin, active β-catenin, **(B)** GSK-3β, and its phosphorylated protein (p-GSK-3β) were determined using a western blot assay (left panel). Statistical results were analyzed using the Mann–Whitney test (n = 5, middle and right panels). USP5, ubiquitin-specific protease 5; EMT, epithelial–mesenchymal transition; GSK, glycogen synthase kinase.

Since the Wnt/β-catenin signaling pathway is an important regulator of EMT in physiological and pathophysiological processes (Sun et al., 2019), we evaluated the effect of USP5 on the cellular levels of β-catenin in H1299 and 95-D cells. The western blot results indicated that knocking down USP5 expression in H1299 and 95-D cells significantly reduced both β-catenin and active β-catenin protein levels compared to those in the control group (**Figure 5A**). The ubiquitination and protein stability assay showed that the ubiquitination of β-catenin increased in H1299-shUSP5 cells (**Figure S3A**), and the levels of β-catenin and active β-catenin proteins were not reduced when treated with MG132 to inhibit the activity of proteasomes (**Figure S3B**). Meanwhile, the levels of GSK-3β protein and its phosphorylated protein, which are negative regulators of the Wnt/β-catenin signaling pathway and located upstream of β-catenin, were significantly increased in both H1299-sh*USP5* and 95-D-sh*USP5* cells compared to those in -shNC cells (**Figure 5B**). Taken together, these findings indicated that USP5 prevented β-catenin degradation by inhibiting its ubiquitination and regulated the Wnt/β-catenin signaling pathway to promote EMT in NSCLC cells.

### Correlation Between the USP5/β-Catenin Axis and EMT in NSCLC Tissues

**Figure 6:**
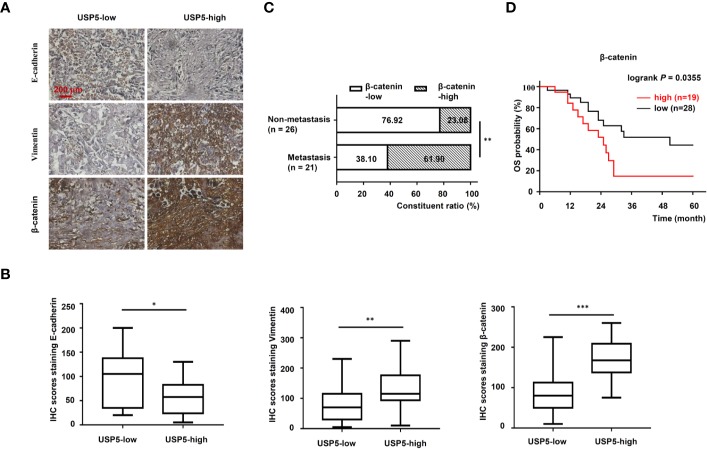
Correlations among USP5 and EMT markers and clinical significance of β-catenin in NSCLC tissues. **(A)** Representative IHC staining for E-cadherin, vimentin, and β-catenin in NSCLC tissues, which were divided into USP5 high-expression and USP5 low-expression subgroups. **(B)** Correlations among USP5 and IHC scores of E-cadherin, vimentin, and β-catenin were analyzed. Statistical results were analyzed using the Mann–Whitney test. **(C)** Comparison of metastatic status in 47 patients with NSCLC with different levels of β-catenin expression. Statistical analyses were performed using the χ^2^ test. **(D)** Kaplan–Meier analysis of OS in patients with NSCLC based on β-catenin expression. Statistical analysis was performed using a log-rank test. ^*^p < 0.05, ^**^p < 0.01, ^***^p < 0.001. USP5, ubiquitin-specific protease 5; EMT, epithelial–mesenchymal transition; NSCLC, non-small cell lung cancer; IHC, immunohistochemistry; OS, overall survival.

To further determine the correlation between the USP5/β-catenin axis and EMT, 47 NSCLC tissue samples were reanalyzed based on IHC tests. A schematic representation of EMT markers and β-catenin expression in NSCLC tissues is shown in **Figure 6A**. The H-scores of EMT markers and β-catenin in USP5-low tissues versus USP5-high tissues were compared. The statistical results indicated a significant increase in vimentin and β-catenin expression among USP5-high tissues compared with that in USP5-low tissues (**Figure 6B**). The E-cadherin expression in USP5-high tissues was markedly decreased.

Furthermore, the correlation between β-catenin expression and metastatic status in 47 patients with NSCLC was analyzed. These patients were classified into β-catenin-high or β-catenin-low subgroups based on the cutoff value (120) of the IHC score. As shown in **Figure 6C**, USP5 expression levels were significantly correlated with tumor metastasis in patients with NSCLC. Kaplan–Meier survival analysis revealed that patients in the β-catenin-high subgroup had significantly worse outcomes compared with those of patients in the β-catenin-low subgroup (**Figure 6D**). This indicated that β-catenin expression was associated with poor prognosis in patients with NSCLC.

## Discussion

NSCLC remains one of the most lethal cancers worldwide, and metastasis is the primary factor that leads to NSCLC mortality (Waqar et al., 2018). Multiple molecular interactions regulated by complex key genes are involved in the process of NSCLC metastasis. Hence, there is an urgent need to identify the regulatory mechanisms of these key genes, providing promising strategies for precise treatment of patients with NSCLC. In the present study, we revealed the correlation of USP5 with the metastasis and prognosis of NSCLC. We then confirmed the role of USP5 in the migration and invasion of NSCLC cells. Furthermore, the underlying molecular mechanism of USP5 that induces EMT through the GSK-3β-mediated Wnt/β-catenin pathway was disclosed. The correlation between the USP5/β-catenin axis and EMT was validated in NSCLC tissues.

Accumulating evidence indicates that aberrant expression or function of DUBs in patients with cancer represents molecular signatures that not only account for tumor recurrence, but are also involved in tumor invasion and metastasis (Yuan et al., 2018). USP5 has been reported to play vital roles in tumor progression in various cancers, including pancreatic cancer, liver cancer, and colorectal cancer (Liu et al., 2017; Li et al., 2017; Xu et al., 2019). However, the relationship between USP5 and NSCLC metastasis remains unknown. Herein, we found that USP5 is significantly highly expressed in NSCLC tissues compared to that in adjacent normal tissues, and high expression of USP5 is significantly associated with poor prognosis in patients with NSCLC who accounted for 47 NSCLC tissues and 2,404 clinical samples from two public datasets. These findings were consistent with prior reports of high expression (85/116) of USP5 protein detected in NSCLC samples and of patients with NSCLC with high USP5 expression exhibiting worse OS (Ma et al., 2018). In addition, we are the first to find that high USP5 expression is significantly associated with tumor metastasis in NSCLC, endowing USP5 with a new function in tumor progression. It has been reported that USP5 is highly expressed and stabilizes SLUG, which is the key transcription factor of EMT, promoting malignant progression in hepatocellular carcinoma cells (Meng et al., 2019). GSK3β-pSer9 has been reported to positively regulate the expression of SLUG in NSCLC (Kao et al., 2014). Herein, we revealed that USP5 downregulates the expression of GSK3β-pSer9 in NSCLC cells, indicating that USP5 may not indirectly regulate SLUG by GSK3β phosphorylation, likely owing to its direct stabilization of SLUG in NSCLC. Meanwhile, USP5 was found to promote EMT, invasion, and migration of NSCLC cells, and its expression is correlated with EMT and metastasis in NSCLC tissues.

Distant metastasis involves the separation of tumor cells from their primary location, which is mainly induced by EMT, their invasion in the circulatory or lymphatic systems, and their establishment at a new location (Mehes et al., 2001). Among them, EMT, characterized by the downregulation of epithelial cell markers (E-cadherin and claudin-1) and upregulation of mesenchymal cell markers (N-cadherin and vimentin) (Polyak and Weinberg, 2009), is an early event in tumor metastasis. However, the relationship between USP5 and EMT in NSCLC cells remains unclear. Here, we found that USP5 resulted in an increase in E-cadherin expression and decreases in N-cadherin and vimentin expression, inhibiting invasion and migration in NSCLC cells. These data indicated that USP5 promotes NSCLC metastasis by inducing EMT.

EMT is triggered by many signaling molecules, which interplay with each other to form a crosstalk network. TGF-β signaling, which acts *via* Smad proteins with intrinsic receptor tyrosine kinase activity, is one of the most important pathways of EMT (Choi et al., 2019). In addition, bone morphogenetic protein, Notch, and Hedgehog activated by various dynamic stimuli from the local microenvironment are also motivators of EMT (Gonzalez and Medici, 2014). In the present study, we found that knocking down USP5 in NSCLC cells decreases the levels of β-catenin protein and active β-catenin protein, increases the ubiquitination of β-catenin, and increases the levels of GSK-3β protein and its phosphorylated protein, which are crucial regulators of Wnt/β-catenin signaling. The Wnt/β-catenin signaling pathway is another important activator of EMT. The Wnt ligands bind to Fz receptors and LRP5/6 coreceptors, inhibiting the activity of the destruction complex including GSK3β, then allowing accumulation and translocation of β-catenin to the nucleus, leading to the transcription of many targeted genes associated with EMT, invasion, and migration (Yao et al., 2011; Martinez-Ramirez et al., 2017). Therefore, considering such an important effect of β-catenin, the expression of β-catenin is highly expressed in patients with USP5-high NSCLC and correlated with tumor metastasis. Along with the decrease in β-catenin expression by knocking down USP5, it is suggested that these beneficial effects of USP5 are involved in the upregulation of Wnt/β-catenin signaling. It has been reported that USP5 promotes tumor proliferation and tumorigenesis through the deubiquitination of histone deacetylase 2 (HDAC2) (Du et al., 2019) and β-catenin (Ma et al., 2018). Meanwhile, HDAC2, a histone deacetylase, decreases GSK-3β protein (Wang et al., 2016), likely by deacetylating its histone proteins, hence impairing its gene transcription. Taken together, it is indicated that USP5 regulates Wnt/β-catenin signaling directly *via* deubiquitinated β-catenin and indirectly *via* the stabilization of HDAC2 or other related proteins that may activate these signaling pathways.

## Conclusion

In summary, our results indicate that USP5 is significantly associated with the metastasis and prognosis of NSCLC. The present data suggest that USP5 regulates EMT by Wnt/β-catenin signaling in NSCLC cells, and there are correlations among USP5/β-catenin, EMT, and metastasis in NSCLC tissues. Moreover, USP5 intervention by shRNA knockdown has been reported to prevent the metastasis of hepatocellular carcinoma *in vivo* (Meng et al., 2019). Therefore, USP5 may serve as a novel prognostic biomarker and provide a potential antimetastatic therapeutic target for the treatment of NSCLC.

## Data Availability Statement

The data analyzed in this study was obtained from [R2 Genomics Analysis and Visualization Platform; Kaplan Meier-plotter dataset; GEPIA dataset], the following licenses/restrictions apply [No restrictions]. Requests to access these datasets should be directed to [XD, aladdine@163.com].

## Ethics Statement

The studies involving human participants were reviewed and approved by The Ethics Committee of the Affiliated Hospital of Jiangsu University; Medical College of Jiangsu University. The patients/participants provided their written informed consent to participate in this study.

## Author Contributions

XD and WWa conceived and designed this study. SX, ZiW, GL, JS, LX, and XW performed the experiments and analyzed the data. SX and GL wrote the manuscript. WWu, XJ, ZhW, and CT participated in data collection of clinical parameters. All authors read and approved the manuscript for publication.

## Funding

This work was supported by grants from the National Natural Science Foundation of China (Grant No. 81902320 and 81703532), Jiangsu Pharmaceutical Association (Grant No. A201614), Science and Technology Funds of Suzhou Municipality (Grant No. SS201848), and Program of Youth Technology and Education Commission of Suzhou Municipality (Grant No. KJXW2017033). The funders had no involvements in the study design, data collection, data analysis, or writing of the paper.

## Conflict of Interest

The authors declare that the research was conducted in the absence of any commercial or financial relationships that could be construed as a potential conflict of interest.
