# Corrigendum: USP5 Promotes Metastasis in Non-Small Cell Lung Cancer by Inducing Epithelial-Mesenchymal Transition *via* Wnt/β-Catenin Pathway

**DOI:** 10.3389/fphar.2020.00948

**Published:** 2020-06-24

**Authors:** Sudong Xue, Wei Wu, Ziyan Wang, Guangxian Lu, Jiantong Sun, Xing Jin, Linjun Xie, Xiaoyu Wang, Caihong Tan, Zheng Wang, Wenjuan Wang, Xinyuan Ding

**Affiliations:** ^1^ Department of Pharmacy, the Affiliated Suzhou Hospital of Nanjing Medical University, Suzhou, China; ^2^ Department of Pharmacy, Zhongshan Hospital, Fudan University, Shanghai, China; ^3^ Department of Pharmacy, The Affiliated Hospital of Jiangsu University, Zhenjiang, China; ^4^ Comprehensive Breast Health Center, Ruijin Hospital, Shanghai Jiao Tong University School of Medicine, Shanghai, China; ^5^ Department of Pharmacy, The Children's Hospital of Soochow University, Suzhou, China

**Keywords:** non-small cell lung cancer, metastasis, epithelial-to-mesenchymal transition, ubiquitin-specific protease 5 (USP5), β-catenin

The correspondence order was incorrect in the original article. The lead contact author should be Xinyuan Ding.

The authors apologize for this error and state that this does not change the scientific conclusions of the article in any way. The original article has been updated.

