# Supplementary figures and images for: USP5 Promotes Metastasis in Non-Small Cell Lung Cancer by Inducing Epithelial-Mesenchymal Transition via Wnt/β-Catenin Pathway
Source: Front Pharmacol. 2020 May 8;11:668. doi: 10.3389/fphar.2020.00668 (PMC7236764; doi:10.3389/fphar.2020.00668)

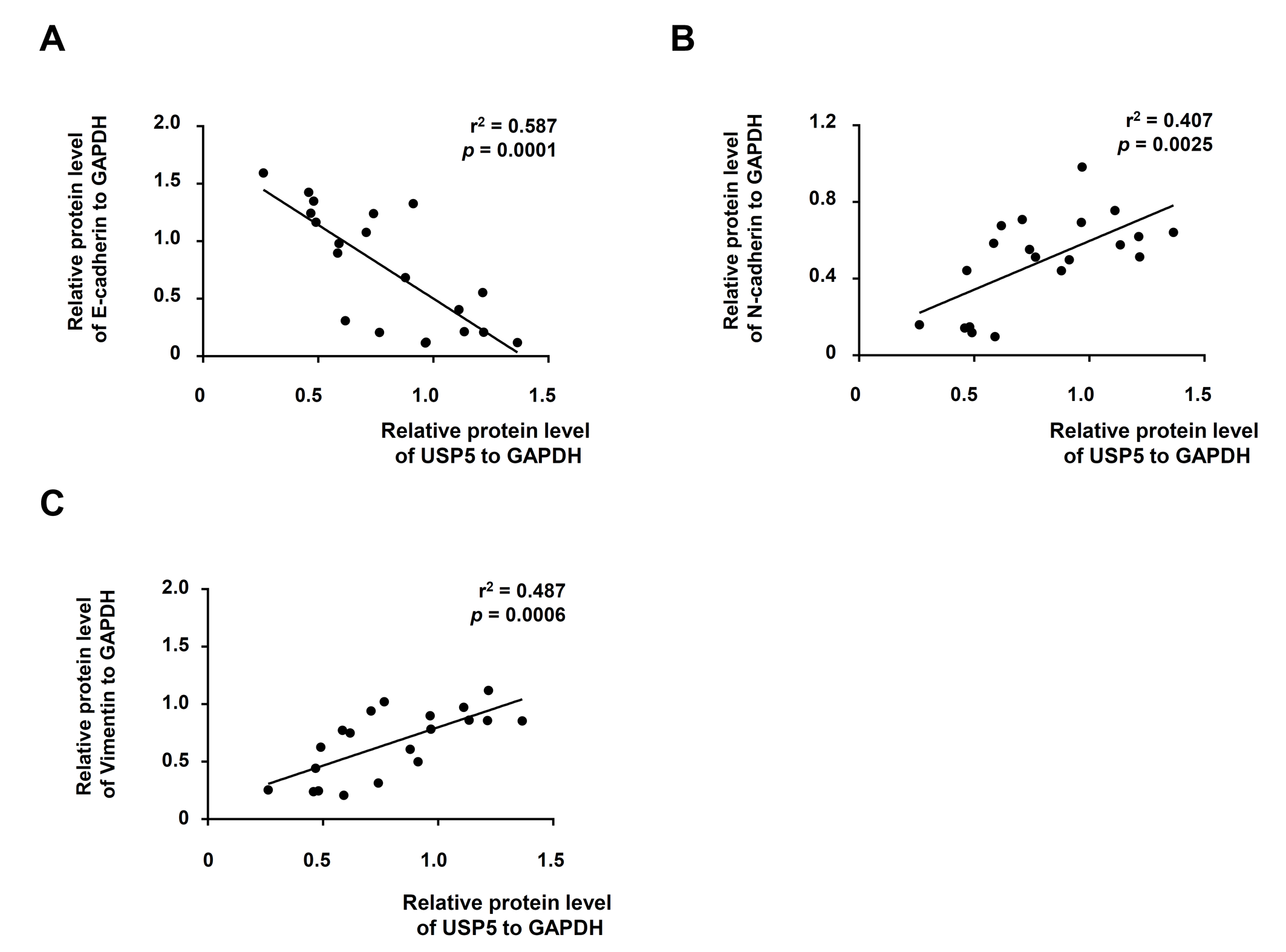

Supplement: Supplementary file 1 [file Image_1.tif]

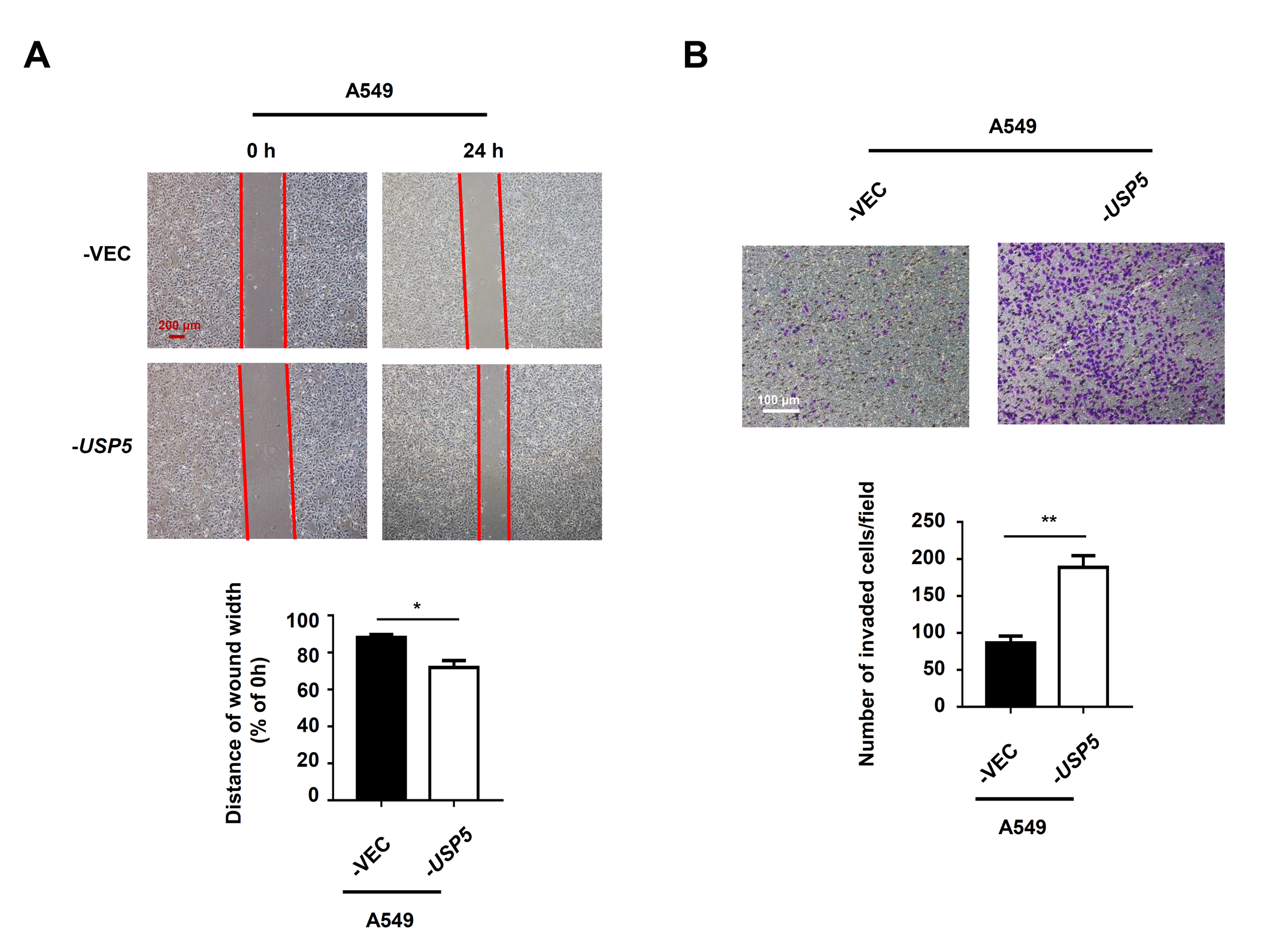

Supplement: Supplementary file 2 [file Image_2.tif]

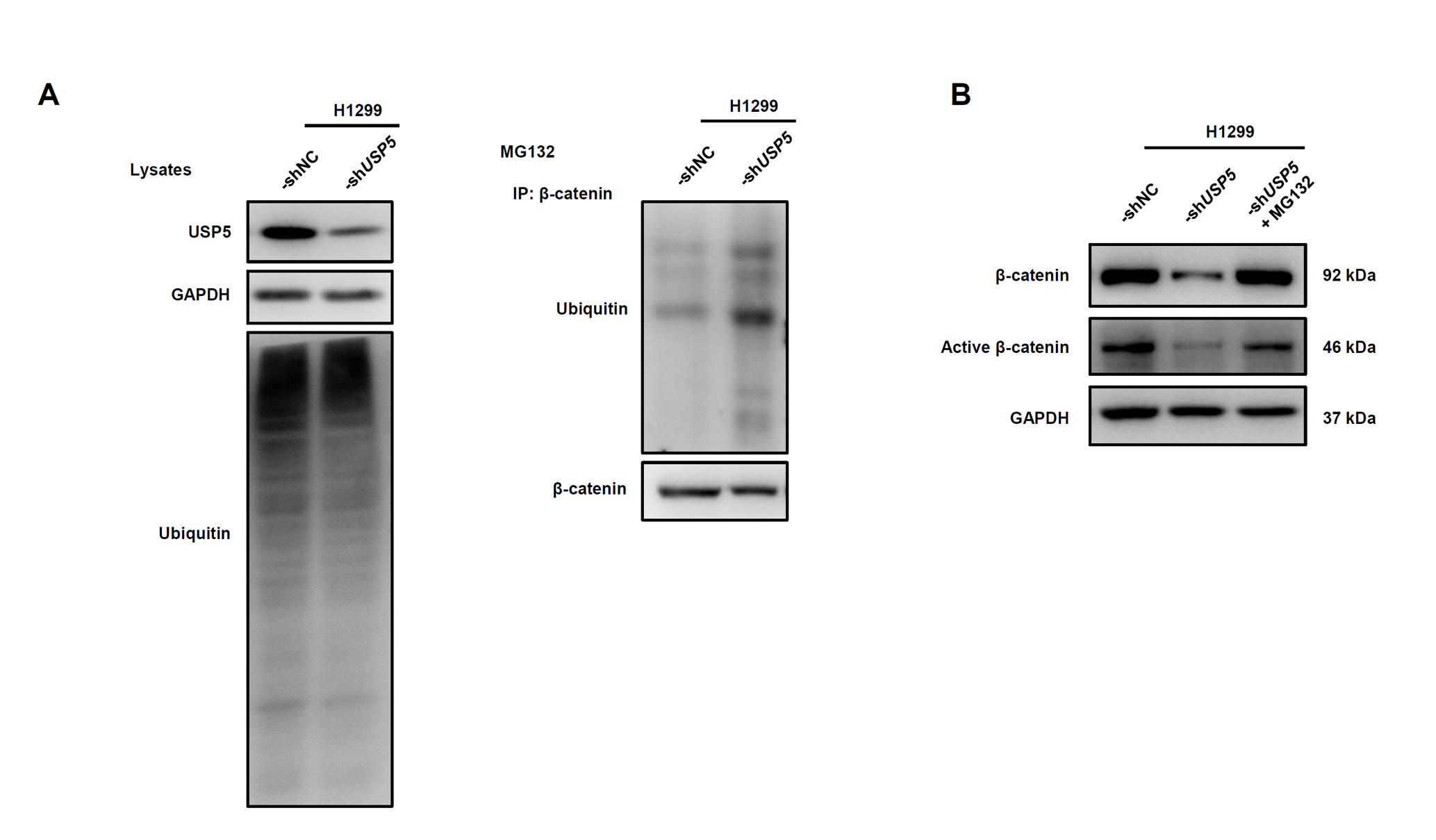

Supplement: Supplementary file 3 [file Image_3.tif]
