# Supplementary material for: USP5 Promotes Metastasis in Non-Small Cell Lung Cancer by Inducing Epithelial-Mesenchymal Transition via Wnt/β-Catenin Pathway
Source: Front Pharmacol. 2020 May 8;11:668. doi: 10.3389/fphar.2020.00668 (PMC7236764; doi:10.3389/fphar.2020.00668)
Supplement: Supplementary file 4 [file DataSheet_1.docx]

**Supplementary Figure**

**Supplementary Figure S1.** Relationships among USP5 and EMT markers. Correlational analysis of USP5 levels (USP5/GAPDH) and **(A)** E-cadherin, **(B)** N-cadherin, and **(C)** vimentin levels (protein levels/GAPDH levels) in NSCLC cells. USP5, ubiquitin-specific protease 5; EMT, epithelial–mesenchymal transition; GAPDH, glyceraldehyde 3-phosphate dehydrogenase; NSCLC, non-small cell lung cancer

**Supplementary Figure S2.** USP5 stabilizes the expression of β-catenin by deubiquitinating its protein. **(A)** Knockdown of USP5 promotes the ubiquitination of β-catenin in H1299 cells. **(B)** Proteasome inhibitor MG132 significantly inhibits the degradation of β-catenin in H1299 cells. USP5, ubiquitin-specific protease 5

**Supplementary Figure S3.** USP5 stabilized the expression of β-catenin by deubiquitinating its protein. **(A)** Knockdown of USP5 promoted the ubiquitination of β-catenin in H1299 cells. **(B)** Proteasome inhibitor MG132 significantly inhibited the degradation of β-catenin in H1299 cells. USP5, ubiquitin-specific protease 5

**Supplementary Table**

**Table S1.** Primers used in real-time PCR.

| **Primer name** | **Sequence (5′- 3′)** |
| --- | --- |
| human *USP5* | GCTGCTGTCAGTATTACCGAC |
|  | AAAGCCCAGAAACGTGTTCATA |
| human *GAPDH* | GGAAGATGGTGATGGGATT |
|  | GGATTTGGTCGTATTGGG |
| human *CDH1*(E-cadherin) | ATTTTTCCCTCGACACCCGAT |
|  | TCCCAGGCGTAGACCAAGA |
| human *VIM* (vimentin) | AGTCCACTGAGTACCGGAGAC |
|  | CATTTCACGCATCTGGCGTTC |

PCR, polymerase chain reaction; USP5, ubiquitin-specific protease 5; GAPDH, glyceraldehyde 3-phosphate dehydrogenase; CDH1, cadherin 1; VIM, vimentin.

**Table S2.** Associations among USP5 expression and clinicopathological characteristics in 509 patients with lung adenocarcinoma.

| Clinical pathologic characteristics | | Case No. | USP5 expression | | *p* |
| --- | --- | --- | --- | --- | --- |
|  |  |  | low | high |  |
| Total cases | | 509 | 284 | 225 |  |
| Gender | |  |  |  |  |
|  | Male | 237 | 118 | 119 | 0.0109^*^ |
|  | Female | 272 | 166 | 106 |  |
| Age (years) | |  |  |  |  |
|  | <60 | 373 | 210 | 163 | 0.7042 |
|  | ≥60 | 136 | 74 | 62 |  |
| TNM stage | |  |  |  |  |
|  | I-II | 398 | 230 | 168 | 0.9565 |
|  | III-IV | 111 | 54 | 57 |  |
| Metastasis | |  |  |  |  |
| Metastasis | | 186 | 87 | 99 | 0.0019^**^ |
| Non-metastasis | | 323 | 197 | 126 |  |

USP5, ubiquitin-specific protease 5; TNM, tumor, node, metastasis. Analysis by χ^2^ test, ^*^*p* < 0.05, ^**^*p* < 0.01 values are set for highly significant differences.
